# Type 2 diabetes: is there any relation between poor control and bone metabolism?

**DOI:** 10.1186/1758-5996-7-S1-A60

**Published:** 2015-11-11

**Authors:** André G Daher Vianna, Laiza Tabisz, Claudio Silva de Lacerda, Luciana Muniz Pechmann, Michelle Garcia Polesel, Emerson Cestari Marino, Fellype Barreto

**Affiliations:** 1 Hospital Da Cruz Vermelha, Curitiba, Brazil

## Background

Numerous evidences suggest that there is a relation between glycemic control and bone fractures in type 2 diabetes individuals. Poorly controlled patients with high blood sugar levels could show higher risk of osteoporosis and/or injuries.

## Objective

This study aims to compare the levels of glycemic control with the serum levels of bone formation and resorpotion markers and the bone mineral density (BMD) in female type 2 diabetes individuals, after menopause.

## Materials and methods

A cross sectional evaluation of 41 female type 2 diabetes individuals was performed. Mean age was 62±5.91 yrs. (mean±SD), time of T2D diagnosis 10.15 ±6.61 yrs., BMI=32.76±6.32. All individuals only used metformin as the anti-diabetic treatment. The glycated hemoglobin (HbA1c) levels were compared to the bone formation markers P1NP and osteocalcin (OC), plus the bone resorption marker CTX as well as lumbar spine, femoral neck and total femoral BMD. Pearson correlation analysis was applied for the total sample, plus comparison between the mean of the groups comprised of quartile 1 (Q1; better control) of HbA1c and quartile 4 (Q4; worse control), through t-test.

## Results

The mean of HbA1c was significantly different between the groups Q1 and Q4 (6.7 ±0.001 vs. 8.1 ±0.003% (p<0.001)). Neither significant correlations nor differences between groups Q1 and Q4 were found related to the HbA1c and P1NP levels (r=0.07; p=0.908), OC (r=-0.12; p=0.388), CTX (r=-0.02; p=0.853), lumber spine BMD(r=0.160; p=0.671), femoral neck BMD (r=0.08; p=0.822) or total femoral BMD (r=0.10 p=0.575).

## Conclusion

We found no significant correlation between levels of glycated hemoglobin and bone formation and resorpotion markers and bone mineral density. There was no difference even when the extreme glycemic control groups were compared.

